# Effect of sustained decreases in sedentary time and increases in physical activity on liver enzymes and indices in type 2 diabetes

**DOI:** 10.3389/fendo.2024.1393859

**Published:** 2024-05-24

**Authors:** Jonida Haxhi, Martina Vitale, Lorenza Mattia, Chiara Giuliani, Massimo Sacchetti, Giorgio Orlando, Carla Iacobini, Stefano Menini, Silvano Zanuso, Antonio Nicolucci, Stefano Balducci, Giuseppe Pugliese

**Affiliations:** ^1^ Department of Clinical and Molecular Medicine, University of Rome La Sapienza, Rome, Italy; ^2^ Diabetes Unit, Sant’Andrea University Hospital, Rome, Italy; ^3^ Metabolic Fitness Association, Rome, Italy; ^4^ Department of Human Movement and Sport Sciences, University of Rome ‘Foro Italico’, Rome, Italy; ^5^ Research Centre for Musculoskeletal Science and Sports Medicine, Department of Life Sciences, Faculty of Science and Engineering, Manchester Metropolitan University, Manchester, United Kingdom; ^6^ Center for Applied Biological and Exercise Sciences, Faculty of Health and Life Sciences, Coventry University, Coventry, United Kingdom; ^7^ Centre for Human Performance and Sport, University of Greenwich, London, United Kingdom; ^8^ Center for Outcomes Research and Clinical Epidemiology (CORESEARCH), Pescara, Italy; ^9^ Department of Clinical Pharmacology and Epidemiology, Consorzio Mario Negri Sud, Santa Maria Imbaro, Italy

**Keywords:** type 2 diabetes, nonalcoholic fatty liver disease, liver enzymes, physical activity, sedentary behavior

## Abstract

**Background:**

Current guidelines for nonalcoholic fatty liver disease (NAFLD) recommend high volumes and/or intensities of physical activity (PA), the achievement of which generally requires participation in supervised exercise training programs that however are difficult to implement in routine clinical practice. Conversely, counselling interventions may be more suitable, but result in only modest increases in moderate-to-vigorous-intensity PA (MVPA). This study assessed whether a counseling intervention for increasing PA and decreasing sedentary time (SED-time) is effective in improving NAFLD markers in people with type 2 diabetes.

**Methods:**

Three-hundred physically inactive and sedentary patients were randomized 1:1 to receive one-month theoretical and practical counseling once-a-year (intervention group) or standard care (control group) for 3 years. Aspartate aminotransferase (AST), alanine aminotransferase (ALT), and γ-glutamyltranspeptidase (γGT) levels were measured and fatty liver index (FLI), hepatic steatosis index (HSI), and visceral adiposity index (VAI) were calculated. Total PA volume, light-intensity PA (LPA), moderate-to-vigorous-intensity PA (MVPA), and SED-time were objectively measured by an accelerometer.

**Results:**

Throughout the 3-year period, NAFLD markers did not change in the control group, whereas ALT, γGT, FLI, and HSI decreased in the intervention group, with significant between-group differences, despite modest MVPA increases, which however were associated with larger decrements in SED-time and reciprocal increments in LPA. Mean changes in NAFLD markers varied according to quartiles of (and correlated with) changes in MVPA (all markers) and SED-time, LPA, and PA volume (ALT, γGT, and HSI). Mean changes in MVPA or PA volume were independent predictors of changes in NAFLD markers. When included in the models, change in cardiorespiratory fitness and lower body muscle strength were independently associated with some NAFLD markers.

**Conclusion:**

A behavior change involving all domains of PA lifestyle, even if insufficient to achieve the recommended MVPA target, may provide beneficial effects on NAFLD markers in people with type 2 diabetes.

## 1 Introduction

Nonalcoholic fatty liver disease (NAFLD) encompasses a broad spectrum of hepatic abnormalities, from simple steatosis to nonalcoholic steatohepatitis (NASH) and cirrhosis (1), and is associated with an increased risk of developing hepatocellular carcinoma (2) and extrahepatic disorders such as cardiovascular disease (3). During the last decades, NAFLD has become the most common chronic liver disease worldwide, due to the ongoing epidemics of obesity and type 2 diabetes (4). Both these conditions are strongly associated with (5, 6) and accelerate progression of (7) NAFLD, which is in fact considered as the hepatic expression of the metabolic syndrome (8). For this reason, an international panel of experts has recently proposed to use the term metabolic dysfunction-associated fatty liver disease instead of NAFLD (9).

Current guidelines recommend, in persons with excess adiposity, a weight loss at least 5% and preferably ≥10% through an energy deficit of 500-1000 kcal with adoption of healthy eating patterns and adherence to physical activity (PA) (10–12). Regarding PA, it is advised to participate in a structured exercise program consisting of 3-5 sessions per week (10, 11), with a total duration of 150-300 min of moderate-intensity or, better, 75-150 min of vigorous-intensity aerobic exercise and eventual addition of resistance exercise training (12). Recommendations on PA are based on the evidence from studies (13–16) and meta-analyses (17–20) that engagement in aerobic and/or resistance exercise programs, generally supervised, is associated with beneficial effects on NAFLD/NASH, with significant decreases in intrahepatic fat accumulation as well as in liver enzymes and indices of steatosis and visceral adiposity. Of note, these effects were independent of weight loss (13–20), though losing weight was associated with greater benefits (18, 21, 22) with a dose-response relationship (23). Moreover, these studies were conducted either in the general population or in people with overweight/obesity and/or NAFLD/NASH, which eventually included individuals with type 2 diabetes.

Unfortunately, adherence to PA recommendation is generally poor (24), especially in people with type 2 diabetes (25), pointing to the need for targeted interventions. However, supervised exercise programs, though very effective in favoring the achievement of the high volumes and/or intensities recommended by guidelines (26), are difficult to implement in routine clinical practice. In contrast, counselling interventions may be more suitable for producing a sustained behavior change, but result in only modest increases in moderate-to-vigorous-intensity PA (MVPA) (27–29), which may be insufficient to improve NAFLD. However, sedentary time (SED-time) was found to be associated with NAFLD, independently of MVPA (30, 31), thus suggesting that targeting also this domain of PA behavior by recommending substitution or interruption with time spent in light-intensity PA (LPA) might be effective in ameliorating NAFLD. In the Italian Diabetes and Exercise Study_2 (IDES_2), a counseling intervention targeting both MVPA and SED-time resulted in only modest increases in MVPA (6.4 min·day-1), but larger (0.8 hours·day-1) decreases in SED-time and reciprocal increases in LPA, thus substantially contributing to the 3.3 metabolic equivalents (METs)-hour·week-1 increment in total PA volume (32). These changes were associated with clinically meaningful improvements in physical fitness and glycemic and blood pressure control, with no significant effect on indices of adiposity or lipid profile (32).

This *post hoc* analysis of the IDES_2 was aimed at evaluating the impact of the counseling intervention and the relative contribution of changes in MVPA and SED-time/LPA on NAFLD markers in people with type 2 diabetes.

## 2 Materials and methods

Design and methods have been detailed elsewhere (32, 33) and will be briefly reported here.

### 2.1 Design

The IDES_2 was an open-label, assessor-blinded, parallel, superiority randomized clinical trial that assessed the efficacy of a behavioral intervention in increasing daily PA and reducing SED-time over a 3-year follow-up in individuals with type 2 diabetes.

### 2.2 Participants

Inclusion criteria were type 2 diabetes of at least one-year duration, age 40-80 years, body mass index (BMI) 27-40 kg/m^2^, physically inactivity (i.e., insufficient amounts of PA according to current guidelines) and sedentary lifestyle (i.e., more than 8 hours/day spent in any waking behavior characterized by an energy expenditure ≤1.5 metabolic equivalents while in a sitting or reclining posture) for at least 6 months, ability to walk 1.6 Km without assistance, and eligibility after cardiologic evaluation. Exclusion criteria were conditions limiting or contraindicating PA, affect conduct of the trial, reduce lifespan, and/or affect the safety of intervention.

### 2.3 Randomization and blinding

Three-hundred individuals with type 2 diabetes were recruited in three tertiary referral, outpatients Diabetes Clinics in Rome and randomized 1:1 to either an intervention (INT) group, receiving theoretical and practical exercise counselling, or a control (CON) group, receiving only general physician recommendations. Randomization was stratified by center and, within each center, by age < versus ≥65 years and non-insulin versus insulin treatment, using a permuted-block randomization software.

Participants from both groups received the same treatment regimen, including dietary prescription, to achieve glycemic, lipid, blood pressure (BP), and body weight targets, according to current guidelines (34). Dietary and pharmacological treatment was adjusted at each visit using a pre-specified algorithm.

Physicians, exercise specialists, and participants were not blinded, whereas assessors of accelerometer/diary and biochemical parameters were blinded to group assignment.

### 2.4 Intervention

Participants in the INT group were engaged in a one-month theoretical and practical counselling, each year for three years. Specifically, the intervention consisted of one individual theoretical counselling session plus eight twice-weekly individual theoretical and practical counselling sessions.

The 30-min theoretical, individual, face-to-face counselling session was held by a diabetologist and consisted of seven steps. Each theoretical and practical counselling session was held by a certified exercise specialist. The theoretical part was aimed at improving knowledge of the effects of exercise on health, conditions contraindicating exercise, difference between habitual and occasional exercise, and essential parameters of wellness such as BP, heart rate, and blood glucose. The practical part served to instruct participants to distinguish the different types of exercise, to evaluate exercise intensity, and to monitor and correct blood glucose imbalances during and after the session.

This approach was designed to promote an increase in any kind of PA, based on individual preference, and a decrease in SED-time through a two-step behavior change, i.e. (1) decreasing SED-time by substituting and/or interrupting it with a wide range of LPAs; and (2) gradually increasing the time spent in purposeful MVPA.

### 2.5 Measurements

#### 2.5.1 Liver enzymes and indices of steatosis and visceral adiposity

At baseline and every 4 months thereafter, levels of aspartate aminotransferase (AST), alanine aminotransferase (ALT), and γ-glutamyltranspeptidase (γGT) were measured by the use of standard methods (VITROS 5,1 FS Chemistry System, Ortho-Clinical Diagnostics Inc, Raritan, NJ). Values of the following indices were then calculated using the formulas reported in **Table 1**: fatty liver index (FLI) (35) and hepatic steatosis index (HSI) (36), two validated indices of steatosis, and visceral adiposity index (VAI), a marker of visceral fat distribution and dysfunction (37) that was found to predict liver histology in individuals with NAFLD (38), though not consistently (39). The following cut-off levels for liver enzymes were used (corresponding to the upper limit of laboratory range): 34 IU/L for AST, 55 IU/L for ALT, and 78 IU/L for γGT, whereas FLI, HSI, and VAI values were considered abnormal if ≥60 (intermediate if 30-59), 36, and 1.9, respectively (36, 37, 40).

**Table 1:** Formulas for calculating indices of steatosis and visceral adiposity.

Index	Formula
**FLI**	exp [0.953 × ln(TG) + 0.139 × BMI + 0.718 × ln(γGT) + 0.053 x [WC] − 15.745]/(1 + exp[0.953 x ln(TG) + 0.139 × BMI + 0.718 × ln(γGT) + 0.053 * [WC] - 15.745]) x 100
**HSI**	8 x (ALT/AST) + BMI (+2, if female; +2, if diabetes mellitus)
**VAI**	Men: {WC/39.68 + [(1.88 x BMI)]} x (TG/1.03) x (1.31/HDL) Women: {WC/[36.58 + (1.89 x BMI)]} x (TG/0.81) x (1.52/HDL)

FLI = fatty liver index; TG = triglycerides; γ-GT = γ-glutamyl-transpeptidase; WC = waist circumference; BMI = body mass index; HSI = hepatic steatosis index; AST = aspartate aminotransferase; ALT = alanine aminotransferase; VAI = visceral adiposity index.

#### 2.5.2 Physical activity and sedentary behavior

Total PA volume, time spent in LPA and MVPA, and SED-time were measured by the use of an accelerometer (MyWellness Key, Technogym, Cesena, IT) and a daily diary for non-accelerometer recordable activities. Measurements were obtained at baseline and every 4 months thereafter for seven consecutive days, except for the initial 4 months, during which the device was worn for the entire period.

#### 2.5.3 Physical fitness

At baseline and every year thereafter, participants were evaluated for physical fitness by assessing cardiorespiratory fitness (as maximal oxygen uptake, VO_2max_), upper and lower body muscle strength, and flexibility by maximal treadmill exercise test, isometric test, and bending test, respectively.

#### 2.5.4 Cardiovascular risk factors

At the same time points, the modifiable cardiovascular risk factors hemoglobin A1c, fasting plasma glucose, BMI, waist circumference, triglycerides, total, HDL, and LDL cholesterol, serum creatinine (with calculation of estimated glomerular filtration rate), albumin:creatinine ratio, high-sensitivity C-reactive protein, and systolic and diastolic BP, were measured using standard methods.

### 2.6 Statistical analysis

Mean changes from baseline throughout the three-year follow-up in liver enzymes and indices, PA/SED-time, physical fitness, and cardiovascular risk factors were calculated for participants who completed the study as the mean values of changes from baseline at each time point (i.e., at 4, 8, 12, 16, 20, 24, 28, 32, and 36 months). Between-group differences in mean changes in AST, ALT, γGT, FLI, HSI, and VAI were assessed by Student’s t test; in addition, differences in liver enzymes and indices throughout the three-year follow-up between INT and CON participants were analyzed by generalized linear mixed models for repeated measures.

To describe the relationships of changes in liver enzymes and indices with those in PA/SED-time, irrespective of study arm, the mean values of changes in AST, ALT, γGT, FLI, HSI, and VAI were then stratified by quartiles of changes in SED-time, MVPA, LPA, and PA volume in the whole cohort and data were expressed as mean ± SD and analyzed by one-way ANOVA. Moreover, univariate correlations between changes in AST, ALT, γGT, FLI, HSI, and VAI and those in SED-time, MVPA, LPA, and PA volume were assessed by Pearson correlation coefficient. Finally, multivariable linear regression analyses with stepwise backward selection of variables were applied to assess the independent predictors of changes in liver enzymes and indices over the three-year period. Study arm, age, sex, the baseline value of the dependent variable, and changes in SED-time and MVPA were included as covariates in Model 1. Changes in LPA were substituted for changes in SED-time in Model 2, whereas changes in PA volume were substituted for changes in SED-time and MVPA in model 3, respectively. All the analyses were repeated by including in the models either changes in BMI or, alternatively, waist circumference, HbA_1c_ or VO_2max_ and lower body muscle strength. Additional analyses were run to assess the independent effect of treatment with anti-hyperglycemic agents on changes in liver enzymes and indices.

All the p-values <0.05 were considered statistically significant. Statistical analyses were performed with SPSS version 20 (SPSS Inc., Chicago, IL, USA).

## 3 Results

As previously reported (32), 267 participants completed the study at the final evaluation (CON=134; INT=133), whereas 33 participants (CON=16; INT=17) dropped out for various reasons; of those in the INT group, >90% attended the counselling sessions.

The baseline features of the individuals considered for the present analysis are reported in **Table 2**.

**Table 2:** Baseline clinical features of all participants who completed the study and by arm.

Parameter	All	CON	INT	*P*
**Age, years**	62.2 ± 9.7	62.7 ± 10.0	61.7 ± 9.5	0.378
**Sex, n (%)**				0.861
** Males**	162 (60.7)	82 (61.2)	80 (60.2)	
** Females**	105 (39.3)	52 (38.8)	53 (39.8)	
**Smoking, n (%)**				0.505
** Never**	110 (41.2)	55 (41.0)	55 (41.4)	
** Former**	110 (41.2)	52 (38.8)	58 (43.6)	
** Current**	47 (17.6)	27 (20.1)	20 (15.0)	
**Diabetes duration, years**	10.8 ± 8.2	11.0 ± 7.9	10.6 ± 8.4	0.692
**HBA_1c_, %**	7.4 ± 1.5	7.3 ± 1.4	7.4 ± 1.5	0.548
**FPG, mmol·l^-1^**	7.51 ± 2.57	7.5 ± 2.6	7.5	2.5 ± 0.859
**BMI, kg·m^2^**	29.9 ± 5.2	30.1 ± 5.6	29.5 ± 4.9	0.303
**Waist circumference, cm**	103.4 ± 12.9	103.9 ± 12.7	102.9 ± 13.1	0.529
**Triglycerides, mmol·l^-1^**	1.79 ± 1.41	1.83 ± 1.77	1.75 ± 0.92	0.663
**Total cholesterol, mmol·l^-1^**	4.66 ± 0.98	4.67 ± 1.01	4.66 ± 0.96	0.962
**HDL cholesterol, mmol·l^-1^**	1.23 ± 0.35	1.21 ± 0.35	1.25 ± 0.36	0.373
**LDL cholesterol, mmol·l^-1^**	2.90 ± 0.86	2.91 ± 0.88	2.89 ± 0.84	0.883
**Systolic BP, mmHg**	140.4 ± 20.8	141.3 ± 21.6	139.5 ± 19.9	0.498
**Diastolic BP, mmHg**	82.8 ± 11.9	83.4 ± 13.6	82.1 ± 10.0	0.366
**eGFR, ml·min^-1^·1.73 m^-2^**	86.8 ± 18.6	86.3 ± 18.7	87.4 ± 18.6	0.645
**ACR, mg·g^-1^**	70.2 ± 345.1	47.8 ± 125.3	92.8 ± 472.4	0.288
**hs-CRP, mg·l^-1^**	4.92 ± 8.99	5.01 ± 9.08	4.84 ± 8.93	0.878
**CHD 10-year risk, %,**	20.9 ± 13.7	21.8 ± 14.7	19.9 ± 12.5	0.264
**Fatal CHD 10-year risk, %**	15.3 ± 12.7	16.2 ± 13.7	14.5 ± 11.5	0.292
**Stroke 10-year risk, %**	13.8 ± 12.6	14.6 ± 12.5	13.1 ± 12.6	0.340
**Fatal stroke 10-year risk, %**	2.27 ± 2.47	2.43 ± 2.58	2.10 ± 2.36	0.265
**AST, UI·L^-1^**	27.1 ± 7.6	26.8 ± 7.0	27.3 ± 8.1	0.605
**ALT, UI·L^-1^**	36.6 ± 13.0	36.3 ± 13.1	37.0 ± 12.9	0.659
**γGT, UI·L^-1^**	33.7 ± 20.7	33.8 ± 21.8	33.7 ± 19.5	0.992
**FLI**	65.8 ± 26.4	66.3 ± 26.7	65.3 ± 26.3	0.746
**HSI**	42.9 ± 6.0	43.0 ± 6.3	42.8 ± 5.7	0.758
**VAI**	2.98 ± 2.63	3.10 ± 3.14	2.87 ± 2.00	0.471
**VO_2max_, ml·min^-1^·kg^-1^**	24.7 ± 6.8	24.7 ± 7.3	24.7 ± 6.3	0.947
**Upper body muscle strength, Nm**	248.9 ± 90.1	250.9 ± 97.0	246.8 ± 82.8	0.712
**Lower body muscle strength, Nm**	158.9 ± 59.6	157.5 ± 59.7	160.3 ± 59.6	0.704
**Bending, cm**	17.0 ± 11.5	18.0 ± 12.2	16.0 ± 10.8	0.158
**SED-time, h·day^-1^**	11.6 ± 1.2	10.6 ± 4.9	11.6 ± 4.6	0.081
**MVPA, min·day^-1^**	12.5 ± 4.7	9.3 ± 4.3	10.1 ± 4.1	0.104
**LPA, h·day^-1^**	3.92 ± 1.36	1.34 ± 0.81	1.52 ± 0.76	0.058
**PA volume, METs-hour·week^-1^**	11.1 ± 4.8	11.6 ± 1.1	11.5 ± 1.2	0.456

CON, control group; INT, intervention group; HBA_1c_, hemoglobin A_1c_; FPG, fasting plasma glucose; BMI, body mass index; BP, blood pressure; eGFR, estimated glomerular filtration rate; ACR, albumin:creatinine ratio; hs-CRP, high-sensitivity C reactive protein; CHD, coronary heart disease; AST, aspartate aminotransferase; ALT, alanine aminotransferase; γ-GT, γ-glutamyl-transpeptidase; FLI, fatty liver index; HSI, hepatic steatosis index; VAI, visceral adiposity index; VO_2max_, maximal oxygen uptake; MVPA, moderate-to-vigorous-intensity physical activity; SED-time, sedentary time; LPA, light-intensity physical activity; PA, physical activity; METs, metabolic equivalents.

### 3.1 Effects of intervention on liver enzymes and indices

No between-group differences were detected at baseline either in liver enzymes and indices or in the parameters used for calculating them (**Table 2**). The percentages of participants with elevated liver enzymes were low (2.6% for AST, 7.9% for ALT, and 4.9% for γGT), whereas the percentages of those with abnormal indices were high (86.9% for FLI, of whom 26.2% with intermediate values, 89.1% for HSI, and 59.9% for VAI), with no significant differences between the two groups.

**Figure 1:**
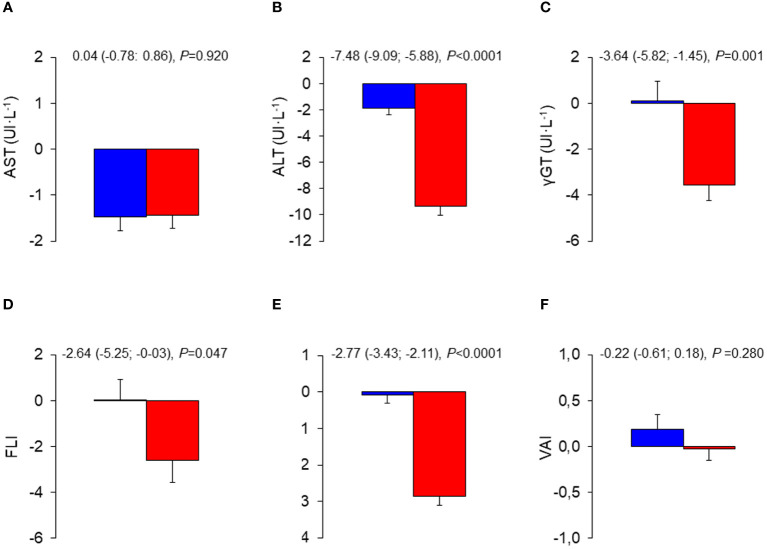
Mean changes from baseline in liver enzymes and indices in the INT and CON group. Baseline to end-of-study changes in AST **(A)**, ALT **(B)**, γ-GT **(C)**, FLI **(D)**, HSI **(E)**, and VAI **(F)** in CON (blue bars) and INT (red bars) participants. AST, aspartate aminotransferase; ALT, alanine aminotransferase; γ-GT, γ-glutamyltranspeptidase; FLI, fatty liver index; HSI, hepatic steatosis index; VAI, visceral adiposity index; CON, control; INT, intervention.

**Figure 2:**
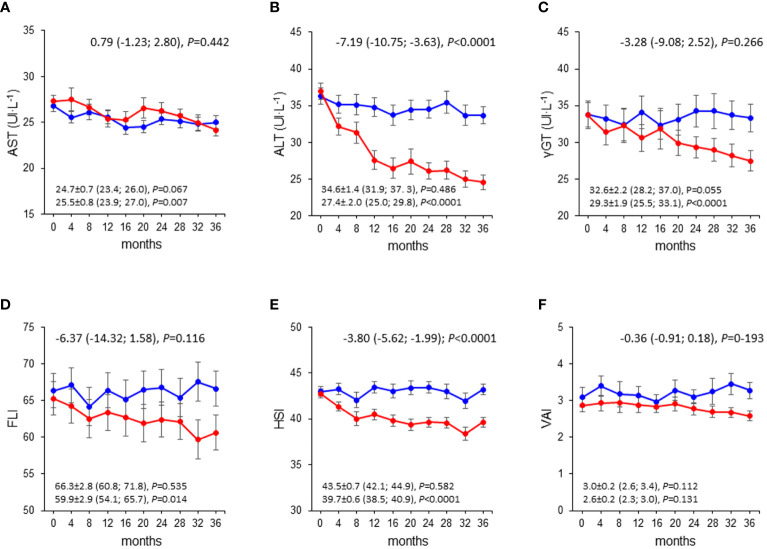
Liver enzymes and indices over the 3-year follow-up in the INT and CON group. Values of AST **(A)**, ALT **(B)**, γ-GT **(C)**, FLI **(D)**, HSI **(E)**, and VAI **(F)** over the 3-year follow-up in CON (blue circles and lines) and INT (red circles and lines) participants. AST, aspartate aminotransferase; ALT, alanine aminotransferase; γ-GT, γ-glutamyltranspeptidase; FLI, fatty liver index; HSI, hepatic steatosis index; VAI, visceral adiposity index; CON, control; INT, intervention.

Mean changes in liver enzymes and indices were negligible in the CON group, whereas those in the INT group indicated substantial decreases in ALT, γGT, FLI, and HSI, but not AST and VAI, with significant between-group differences (**Figure 1**). The analysis of liver enzymes and indices throughout the 3-year follow-up period showed no change in the CON group, and significant decreases in ALT, γGT, FLI, and HSI, but not AST and VAI, in the INT group, with significant between-group differences for ALT and HSI only (**Figure 2**). At end-of-study, the percentages of participants with elevated liver enzyme levels (1.6% for AST, 2,7% for ALT, and 3.5% for γGT) and abnormal indices (84.3% for FLI, of whom 24.7% with intermediate values, and 82.4% for HSI) were lower than at baseline, except for VAI (64.9%). In the CON group, the individuals that became abnormal were more numerous than those that returned normal, whereas the opposite was observed in the INT group (not shown).

### 3.2 Relationships between changes in liver enzymes and indices and changes in PA/SED-time and fitness

Mean changes from baseline in liver enzymes and indices significantly varied according to quartiles of changes in MVPA, with participants falling in quartiles III and IV showing the most marked reductions in these parameters. The same trend was observed according to quartiles of changes in SED-time, LPA, and PA volume, though only for ALT, γGT, and HSI (**Table 3**).

**Table 3:** Mean changes from baseline in liver enzymes and indices according to quartiles of mean changes from baseline in SED-time, MVPA, LPA, or PA volume.

Variables	Quartiles of mean change in SED-time *vs*. baseline				*p*
Mean change in:	I	II	III	IV	
**N**	67	66	68	66	
**SED-time, h·day^-1^**	0.72 ± 0.39	-0.09 ± 0.17	-0.60 ± 0.18	-1.53 ± 0.43	
**(range)**	(1.94; 0.23)	(0.22; -0.33)	(-0.34; -0.99)	(-1.00; -3.08)	
**AST, UI·L^-1^**	-1.25 ± 3.74	-2.05 ± 3.28	-1.26 ± 3.66	-1.26 ± 2.86	0.446
**ALT, UI·L^-1^**	-2.22 ± 7.21	-4.03 ± 6.63	-7.19 ± 7.93	-9.04 ± 6.98	<0.0001
**γGT, UI·L^-1^**	0.90 ± 10.86	-0.50 ± 8.28	-2.77 ± 8.23	-4.51 ± 8.57	0.003
**FLI**	-0.37 ± 9.29	-0.85 ± 12.94	-0.65 ± 9.70	-3.28 ± 11.25	0.388
**HSI**	-0.42 ± 2.98	-0.65 ± 3.17	-1.81 ± 2.82	-2.97 ± 2.65	<0.0001
**VAI**	0.37 ± 1.95	-0.08 ± 1.88	-0.06 ± 1.20	0.11 ± 1.37	0.357
Variables	Quartiles of mean change in MVPA *vs*. baseline				*p*
Mean change in:	I	II	III	IV	
**N**	67	67	67	66	
**MVPA, min·day^-1^**	-3.33 ± 2.17	0.27 ± 0.84	4.02 ± 1.58	14.32 ± 6.59	
**(range)**	(-10.81; -1.09)	(-1.08; 1.87)	(1.88; 7.20)	(7.24; 34.95)	
**AST, UI·L^-1^**	-1.36 ± 3.44	-1.16 ± 4.10	-2.76 ± 3.24	-0.51 ± 2.22	0.001
**ALT, UI·L^-1^**	-0.99 ± 5.62	-4.05 ± 7.51	-8.99 ± 8.10	-8.49 ± 6.20	<0.0001
**γGT, UI·L^-1^**	2.50 ± 6.64	0.57 ± 9.94	-4.07 ± 10.14	-5.94 ± 7.21	<0.0001
**FLI**	2.47 ± 9.48	-0.12 ± 9.56	-3.60 ± 10.90	-3.90 ± 12.30	0.001
**HSI**	0.36 ± 2.91	-1.08 ± 3.14	-2.18 ± 2.66	-2.97 ± 2.53	<0.0001
**VAI**	0.38 ± 2.01	0.32 ± 0.93	-0.42 ± 2.01	0.06 ± 1.19	0.017
Variables	Quartiles of mean change in LPA *vs*. baseline				*p*
Mean change in:	I	II	III	IV	
**N**	66	67	67	67	
**LPA, h·day^-1^**	-0.73 ± 0.41	0.05 ± 0.15	0.55 ± 0.18	1.36 ± 0.50	
**(range)**	(-1.96; -0.20)	(-0.19; 0.29)	(0.31; 0.90)	(0.92; 3.70)	
**AST, UI·L^-1^**	-1.91 ± 3.29	-0.92 ± 3.66	-1.55 ± 3.71	-1.43 ± 2.88	0.409
**ALT, UI·L^-1^**	-2.60 ± 7.76	-4.17 ± 6.47	-6.67 ± 7.86	-8.98 ± 6.98	<0.0001
**γGT, UI·L^-1^**	-0.10 ± 9.99	-0.67 ± 9.97	-1.81 ± 8.21	-4.26 ± 8.27	0.046
**FLI**	-0.13 ± 10.50	-0.53 ± 11.56	-2.19 ± 12.25	-2.24 ± 8.99	0.565
**HSI**	-0.48 ± 3.24	-0.98 ± 3.05	-1.59 ± 2.91	-2.78 ± 2.63	<0.0001
**VAI**	0.09 ± 2.40	0.25 ± 1.19	-0.06 ± 1.56	0.05 ± 1.09	0.740
Variables	Quartiles of mean change in PA volume *vs*. baseline				*p*
Mean change in:	I	II	III	IV	
**N**	66	67	67	67	
**PA volume, METs-hour·week^-1^**	-1.90 ± 1.34	0.22 ± 0.34	1.85 ± 0.57	4.85 ± 1.57	
**(range)**	(-6.57; -0.43)	(-0.35; 0.81)	(0.90; 3.02)	(3.06; 9.14)	
**AST, UI·L^-1^**	-1.45 ± 3.59	-1.62 ± 3.60	-1.70 ± 3.57	-1.04 ± 2.83	0.683
**ALT, UI·L^-1^**	-1.60 ± 7.11	-4.33 ± 7.18	-7.35 ± 7.63	-9.14 ± 6.52	<0.0001
**γGT, UI·L^-1^**	0.97 ± 9.44	-0.25 ± 10.34	-3.25 ± 7.95	-4.30 ± 8.20	0.002
**FLI**	0.25 ± 9.37	0.08 ± 11.61	-2.98 ± 13.26	-2.44 ± 8.45	0.191
**HSI**	-0.36 ± 3.32	-0.57 ± 2.88	-2.05 ± 2.96	-2.85 ± 2.39	<0.0001
**VAI**	0.16 ± 2.42	0.22 ± 1.20	-0.14 ± 1.51	0.09 ± 1.10	0.600

Values are mean ± SD, unless otherwise specified. SED-time, sedentary time; MVPA, moderate-to-vigorous-intensity physical activity; LPA, light-intensity physical activity; PA, physical activity; METs, metabolic equivalents; AST, aspartate aminotransferase; ALT, alanine aminotransferase; γ-GT, γ-glutamyl-transpeptidase; FLI, fatty liver index; HSI, hepatic steatosis index; VAI, visceral adiposity index.

Likewise, at univariate analysis (**Table 4**), mean changes from baseline in ALT, γGT, and HSI significantly correlated with mean changes in SED-time and, inversely, with those in MVPA, LPA, and total PA volume, whereas changes in FLI and VAI correlated significantly only with changes in MVPA.

**Table 4:** Univariate correlation between mean changes from baseline in liver enzymes and indices and those in SED-time, MVPA, LPA, or PA volume.

Change in:	SED-time change		MVPA change		LPA change		PA volume change	
	rho	*p*	rho	*p*	rho	*p*	rho	*p*
**AST**	-0.073	0.233	0.056	0.361	0.049	0.428	0.077	0.212
**ALT**	0.387	<0.0001	-0.519	<0.0001	-0.351	<0.0001	-0.435	<0.0001
**γGT**	0.309	<0.0001	-0.482	<0.0001	-0.251	<0.0001	-0.342	<0.0001
**FLI**	0.062	0.311	-0.212	<0.0001	-0.055	0.370	-0.088	0.152
**HSI**	0.367	<0.0001	-0.447	<0.0001	-0.364	<0.0001	-0.440	<0.0001
**VAI**	0.039	0.526	-0.139	0.023	-0.082	0.182	-0.081	0.188

SED-time, sedentary time; MVPA, moderate-to-vigorous-intensity physical activity; LPA, light-intensity physical activity; PA, physical activity; AST, aspartate aminotransferase; ALT, alanine aminotransferase; γ-GT, γ-glutamyl-transpeptidase; FLI, fatty liver index; HSI, hepatic steatosis index; VAI, visceral adiposity index.

At multivariable linear regression analysis (**Table 5**), change in MVPA, but not change in SED-time (in Model 1) or LPA (in Model 2), was an independent predictor of changes in ALT, γGT, FLI, HSI, and VAI, but not AST. Removal of MVPA change from the models resulted in a significant association of change in SED-time with changes in ALT, γGT, HSI, and VAI or of change in LPA with changes in ALT and VAI (not shown). In Model 3, change in PA volume was an independent predictor of changes in the same parameters as MVPA in Model 1 and 2. In addition, study arm, age, and sex were variably associated with changes in liver enzymes and indices, whereas the baseline value of the dependent variable was significantly associated with its change over the study follow-up, except for HSI. When included in the models, change in BMI was significantly associated with changes in AST, ALT (in Model 2 only), γGT, FLI, and HSI, change in waist circumference was significantly associated with change in FLI, HSI, and VAI, and change in HbA1c was significantly associated with changes in γGT, FLI, HSI, and VAI, without substantially modifying the relationships between changes in MVPA or PA volume with changes in liver enzymes and indices (not shown). Moreover, among the antihyperglycemic agents, only glucagon-like peptide-1 receptor agonists (GLP-1 RAs) and thiazolidinediones (TDZs), which were used in 14 CON versus 20 INT (*P*=0.261) and 33 CON versus 17 INT (*P*=0.013) participants, respectively, at any time during the 3-year period, were independently associated with changes in NAFLD markers, in particular HSI for GLP-1 RAs and VAI for both, without any significant impact on the association between changes in these indices and those in MVPA or PA volume. Finally, when included in the models, change in VO_2max_ was associated with changes in ALT and VAI in Model 1 and 2 and changes in ALT, γGT, and HSI in Model 3, whereas change in lower body muscle strength was an independent predictor of changes in FLI and, in Model 3 only, VAI, without substantially modifying the relationships between changes in MVPA or PA volume with changes in liver enzymes and indices (**Table 6**).

**Table 5:** Independent predictors of mean changes from baseline in liver enzymes and indices.

Model 1												
Dependent variable change	AST		ALT		γGT		FLI		HSI		VAI	
	Beta	*p*	Beta	*p*	Beta	*p*	Beta	*p*	Beta	*p*	Beta	*p*
**Study arm**	–	–	-5.703	<0.0001	–	–	–	–	-2.160	<0.0001	–	–
**Age**	–	–	-0.071	0.071	–	–	-0.151	0.021	–	–	-0.014	0.090
**Sex**	-0.910	0.028	-1.824	0.020	–	–	-2.329	0.076	-0.761	0.023	–	–
**MVPA change**	–	–	-0.251	<0.0001	-0.449	<0.0001	-0.287	0.001	-0.090	<0.0001	-0.031	0.005
**SED-time change**	–	–	–	–	–	–	–	–	–	–	–	–
**Dependent variable baseline**	-0.132	<0.0001	-0.198	<0.0001	-0.134	<0.0001	-0.092	<0.0001	–	–	-0.359	<0.0001
Model 2												
Dependent variable change	AST		ALT		γGT		FLI		HSI		VAI	
	Beta	*p*	Beta	*p*	Beta	*p*	Beta	*p*	Beta	*p*	Beta	*p*
**Study arm**	–	–	-5.703	<0.0001	–	–	–	–	-2.160	<0.0001	–	–
**Age**	–	–	-0.071	0.071	–	–	-0.151	0.021	–	–	-0.014	0.090
**Sex**	-0.910	0.028	-1.824	0.020	–	–	-2.329	0.076	-0.761	0.023	–	–
**MVPA change**	–	–	-0.251	<0.0001	-0.449	<0.0001	-0.287	0.001	-0.090	<0.0001	-0.031	0.005
**LPA change**	–	–	–	–	–	–	–	–	–	–	–	–
**Dependent variable baseline**	-0.132	<0.0001	-0.198	<0.0001	-0.134	<0.0001	-0.092	<0.0001	–	–	-0.359	<0.0001
Model 3												
Dependent variable change	AST		ALT		γGT		FLI		HSI		VAI	
	Beta	*p*	Beta	*p*	Beta	*p*	Beta	*p*	Beta	*p*	Beta	*p*
**Study arm**	–	–	-6.085	<0.0001	–	–	–	–	-2.383	<0.0001	–	–
**Age**	–	–	-0.072	0.070	–	–	-0.156	0.019	–	–	-0.015	0.066
**Sex**	-0.910	0.028	-1.689	0.033	–	–	-2.285	0.085	-0.728	0.033	–	–
**PA volume change**	–	–	-0.512	0.002	-0.891	<0.0001	-0.581	0.015	-0.148	0.037	-0.102	0.001
**Dependent variable baseline**	-0.132	<0.0001	-0.186	<0.0001	-0.133	<0.0001	-0.086	<0.0001	–	–	-0.362	<0.0001

AST, aspartate aminotransferase; ALT, alanine aminotransferase; γ-GT, γ-glutamyl-transpeptidase; FLI, fatty liver index; HSI, hepatic steatosis index; VAI, visceral adiposity index; BMI, body mass index; MVPA, moderate-to-vigorous-intensity physical activity; SED-time, sedentary time; LPA, light-intensity physical activity; PA, physical activity.

**Table 6:** Independent predictors of mean changes from baseline in liver enzymes and indices.

Model 1												
Dependent variable change	AST		ALT		γGT		FLI		HSI		VAI	
	Beta	*p*	Beta	*p*	Beta	*p*	Beta	*p*	Beta	*p*	Beta	*p*
**Study arm**	–	–	-5.087	<0.0001	–	–	–	–	-2.053	<0.0001	–	–
**Age**	–	–	-0.068	0.083	–	–	-0.168	0.011	–	–	-0.016	0.065
**Sex**	-0.963	0.019	-2.100	0.008	–	–	-2.462	0.060	-0.699	0.038	–	–
**MVPA change**	–	–	-0.194	0.003	-0.459	<0.0001	-0.187	0.043	-0.098	<0.0001	–	–
**SED-time change**	–	–	–	–	–	–	–	–	–	–	–	–
**VO_2max_ change**	–	–	-0.296	0.017	–	–	–	–	–	–	-0.056	0.025
**Lower body strength change**	–	–	–	–	–	–	-0.065	0.006	–	–	-0.006	0.069
**Dependent variable baseline**	-0.138	<0.0001	-0.201	<0.0001	-0.132	<0.0001	-0.094	<0.0001	–	–	-0.361	<0.0001
Model 2												
Dependent variable change	AST		ALT		γGT		FLI		HSI		VAI	
	Beta	*p*	Beta	*p*	Beta	*p*	Beta	*p*	Beta	*p*	Beta	*p*
**Study arm**	–	–	-5.087	<0.0001	–	–	–	–	-2.053	<0.0001	–	–
**Age**	–	–	-0.068	0.083	–	–	-0.168	0.011	–	–	-0.016	0.065
**Sex**	-0.963	0.019	-2.100	0.008	–	–	-2.462	0.060	-0.699	0.038	–	–
**MVPA change**	–	–	-0.194	0.003	-0.459	<0.0001	-0.187	0.043	-0.098	<0.0001	–	–
**LPA change**	–	–	–	–	–	–	–	–	–	–	–	–
**VO_2max_ change**	–	–	-0.296	0.017	–	–	–	–	–	–	-0.056	0.025
**Lower body strength change**	–	–	–	–	–	–	-0.065	0.006	–	–	-0.006	0.069
**Dependent variable baseline**	-0.138	<0.0001	-0.201	<0.0001	-0.132	<0.0001	-0.094	<0.0001	–	–	-0.361	<0.0001
Model 3												
Dependent variable change	AST		ALT		γGT		FLI		HSI		VAI	
	Beta	*p*	Beta	*p*	Beta	*p*	Beta	*p*	Beta	*p*	Beta	*p*
**Study arm**	–	–	-5.915	<0.0001	–	–	–	–	-2.348	<0.0001	–	–
**Age**	–	–	-0.067	0.092	–	–	-0.166	0.013	–	–	-0.017	0.045
**Sex**	-0.963	0.019	-2.100	0.009	–	–	-2.624	0.047	-0.742	0.031	–	–
**PA volume change**	–	–	–	–	-0.527	0.025	–	–	–	–	-0.077	0.021
**VO_2max_ change**	–	–	-0.477	<0.0001	-0.469	0.005	-0.337	0.084	-0.136	0.005	–	–
**Lower body strength change**	–	–	–	–	–	–	-0.062	0.013	–	–	-0.007	0.025
**Dependent variable baseline**	-0.138	<0.0001	-0.199	<0.0001	-0.129	<0.0001	-0.093	<0.0001	–	–	-0.365	<0.0001

AST, aspartate aminotransferase; ALT, alanine aminotransferase; γ-GT, γ-glutamyl-transpeptidase; FLI, fatty liver index; HSI, hepatic steatosis index; VAI, visceral adiposity index; MVPA, moderate-to-vigorous-intensity physical activity; SED-time, sedentary time; LPA, light-intensity physical activity; PA, physical activity.

## 4 Discussion

This *post hoc* analysis of the IDES_2 showed that sustained decreases in SED-time and increases in PA promoted by a behavioral counseling were associated with improvements in NAFLD markers, with significant reductions of ALT, γGT, FLI, and HSI, whereas VAI was unchanged, consistent with the lack of effect of the intervention on indices of adiposity such as BMI and waist circumference (32). Though relatively modest, the improvements in NAFLD markers were similar to those reported in previous studies (41, 42) and meta-analyses (17, 19) for supervised exercise programs of shorter duration. In particular, in the previous IDES (42), enzyme levels did not change, whereas FLI and VAI significantly decreased in the supervised exercise intervention group, but not in the control group, in a PA volume-dependent manner.

However, participants in supervised exercise studies were engaged in moderate-to vigorous intensity training or high intensity interval training at least three times a week, thus accumulating large amounts of MVPA, which were shown to be required for obtaining significant decreases in intrahepatic fat accumulation (43). Conversely, in the IDES_2, increments in MVPA were much lower, though sustained over three years, and not sufficient to achieve the recommended target of at least 150-300 min per week. Thus, the novel information provided by our study is that even small increments in MVPA obtained by adopting and maintaining a more active lifestyle can be effective in improving NAFLD markers in individuals with type 2 diabetes, suggesting that “some (MVPA) is better than nothing”. Yet, the findings that participants falling in quartiles III and IV of MVPA change showed the most marked reductions in liver enzymes and indices and that the increase in MVPA was a strong independent predictor of improvements in NAFLD markers confirm the concept that “the more (MVPA) the better”.

Moreover, the modest changes in MVPA were accompanied by larger decrements in SED-time and reciprocal increments in LPA resulting in substantial increases in total PA volume. These changes were also associated with changes in liver enzymes and indices, consistent with previous evidence that sedentary behavior or, inversely, overall PA level are associated with presence of NAFLD or increased liver enzymes (30, 31, 44, 45). These findings suggest that NAFLD may also benefit from reallocation of SED-time to LPA. In fact, though changes in SED-time or LPA were not independent predictors of variations in NAFLD markers, a role for decreases in SED-time through the reciprocal increases in LPA is supported by the independent association of liver enzymes and indices with total PA volume, of which increments in LPA were a main contributor.

Another important finding of this study is that changes in VO_2max_ and lower body muscle strength predicted changes in NAFLD markers beyond changes in PA/SED-time, thus suggesting that the improvements in physical fitness resulting from the behavioral modification provided additional benefits. This is consistent with the observations that cardiorespiratory fitness is associated with presence of NAFLD, as assessed by FLI, independent of MVPA (46), and that baseline VO_2max_ is an independent predictor of reduction in liver fat from a lifestyle intervention in individuals with NAFLD (47). Moreover, changes in BMI and, to a lesser extent, waist circumference were also associated, independent of changes in PA/SED-time, with changes in NAFLD markers, including the indices calculated using these parameters.

Several mechanisms have been hypothesized for explaining the beneficial effects on NAFLD of increasing PA and physical fitness and decreasing SED-time in the absence of significant reductions in total and central fat mass. These mechanisms include amelioration of insulin resistance at both the liver and adipose tissue level and increased mitochondrial biogenesis and capillarization favoring fatty acid uptake, β-oxidation, and triglyceride storage at the muscle level (48). The importance of muscle in the pathogenesis of NAFLD is supported by the inverse association of muscle mass and grip strength with risk of severe NAFLD (49) and the correlation between muscle fat accumulation and NASH severity (50). These mechanisms might be operating also for modest increases in MVPA, provided that they are sustained in the long-term.

The main strength of this study is the evaluation of changes in liver enzymes and indices of steatosis associated with long-term, sustained changes in PA/SED-time, as measured objectively by the use of an accelerometer. Other strengths concern the trial design, including the application of an intervention targeting both PA and SED-time, based on solid theoretical grounds, and using several behavioral change techniques, the specific training of investigators, the long study duration, and the large sample size (32, 33). However, this study has some limitations. First, the use of surrogate measures, such as liver enzymes and indices of steatosis, instead of direct methods, such as the gold standard histology or imaging techniques, does not allow to draw definite conclusions regarding the effect of the intervention on NAFLD/NASH. Second, the lack of data on platelet count did not allow calculation of indices of liver fibrosis. Third, generalization requires further investigation and validation in different cohorts or settings. Fourth, results might have been affected by unmeasured confounders, for instance diet, which was not considered in data analysis, though participants received dietary prescriptions and adherence to diet was verified at intermediate visits.

## 5 Conclusion

This *post hoc* analysis of the IDES_2 showed that, in individuals with type 2 diabetes, a counseling intervention for increasing PA and decreasing SED-time was effective in ameliorating NAFLD, as suggested by the improvements in liver enzymes and indices of steatosis over 3 years. Changes in MVPA, though modest, were the main predictors of changes in these surrogate measures, but also the greater decreases in SED-time contributed to this effect, possibly through the reciprocal increases in LPA that resulted in a relatively large increment in total PA volume. These findings indicate that a behavior change involving all domains of PA lifestyle, even if insufficient to achieve the recommended MVPA target, may also provide beneficial effects on NAFLD/NASH in people with type 2 diabetes, eventually combined with treatment with GLP-1 RAs and/or TDZs.

## Data availability statement

The raw data supporting the conclusions of this article will be made available by the authors, without undue reservation.

## Ethics statement

The studies involving humans were approved by Ethics Committee of Sant’Andrea University Hospital. The studies were conducted in accordance with the local legislation and institutional requirements. The participants provided their written informed consent to participate in this study.

## Author contributions

JH: Conceptualization, Data curation, Formal analysis, Investigation, Validation, Writing – review & editing. MV: Conceptualization, Data curation, Formal analysis, Investigation, Writing – review & editing. LM: Data curation, Formal analysis, Investigation, Writing – review & editing. CG: Data curation, Formal analysis, Investigation, Writing – review & editing. MS: Investigation, Methodology, Resources, Validation, Writing – review & editing. GO: Investigation, Methodology, Resources, Validation, Writing – review & editing. CI: Investigation, Validation, Writing – review & editing. SM: Investigation, Visualization, Writing – review & editing. SZ: Investigation, Methodology, Resources, Validation, Writing – review & editing. AN: Data curation, Formal analysis, Software, Writing – review & editing. SB: Data curation, Formal analysis, Funding acquisition, Investigation, Project administration, Supervision, Writing – review & editing, Resources. GP: Conceptualization, Data curation, Formal analysis, Investigation, Project administration, Supervision, Writing – original draft.

## Group members of Italian Diabetes Exercise Study 2 (IDES_2) Investigators

See **Supplementary Data Sheet 1** for a complete list of the IDES_2 Investigators.
