# Supplementary material for: Effect of sustained decreases in sedentary time and increases in physical activity on liver enzymes and indices in type 2 diabetes
Source: Front Endocrinol (Lausanne). 2024 May 24;15:1393859. doi: 10.3389/fendo.2024.1393859 (PMC11157683; doi:10.3389/fendo.2024.1393859)
Supplement: Supplementary file 1 [file DataSheet_1.docx]

Supplementary Material

**Supplementary file 1: The IDES_2 Investigators**

**Principal Investigator: Giuseppe Pugliese**, MD, PhD, Department of Clinical and Molecular Medicine, University of Rome La Sapienza, and Diabetes Unit, Sant’Andrea University Hospital, Via di Grottarossa, 1035-1039 - 00189 Rome, Italy; Phone: +39-0633775440; Fax: +39-0633776327; E-mail: [giuseppe.pugliese@uniroma1.it](mailto:giuseppe.pugliese@uniroma1.it).

**Co-Investigator: Stefano Balducci**, MD, Metabolic Fitness Association O.N.L.U.S., Via Nomentana, 27 - 00015 Monterotondo, Rome, Italy; Phone +390690080260; Fax: +390690080235; e-mail: [sbalducci@esinet.it](mailto:sbalducci@esinet.it).

**Steering Committee**

**Giuseppe Pugliese**, MD, PhD, Department of Clinical and Molecular Medicine, University of Rome La Sapienza, Via di Grottarossa, 1035-1039 - 00189 Rome, Italy; E-mail: [giuseppe.pugliese@uniroma1.it](mailto:giuseppe.pugliese@uniroma1.it). (Principal Investigator).

**Stefano Balducci**, MD, Metabolic Fitness Association O.N.L.U.S., Via Nomentana, 27 - 00015 Monterotondo, Rome, Italy; E-mail: [sbalducci@esinet.it](mailto:sbalducci@esinet.it). (Co-Investigator)

**Massimo Sacchetti**, PhD, Department of Human Movement and Sport Sciences, University of Rome ‘Foro Italico’, Piazza de Bosis, 15, 00135 Rome, Italy; E-mail: [massimo.sacchetti@uniroma4.it](mailto:massimo.sacchetti@uniroma4.it). (Physical fitness)

**Silvano Zanuso**, PhD, Center for Applied Biological & Exercise Sciences, Faculty of Health & Life Sciences, Coventry University, Priory Street, Coventry, United Kingdom CV1 5FB; E-mail: [szanuso@gmail.com](mailto:szanuso@gmail.com). (Accelerometer)

**Patrizia Cardelli**, PhD, Department of Clinical and Molecular Medicine, University of Rome La Sapienza, and Laboratory of Clinical Chemistry, Sant’Andrea University Hospital, Via di Grottarossa, 1035-1039 - 00189 Rome, Italy; E-mail: [patrizia.cardelli@uniroma1.it](mailto:patrizia.cardelli@uniroma1.it). (Biochemical tests)

**Antonio Nicolucci**, MD, PhD, Center for Outcomes Research and Clinical Epidemiology (CORESEARCH), Via Tiziano Vecellio, 2, 65124 Pescara, Italy**;** E-mail: [nicolucci@coresearch.it](mailto:nicolucci@coresearch.it). (Central randomization and statistical analysis)

**Participating centers**

***Diabetes Clinics***

1. Diabetes Unit, Sant’Andrea University Hospital, Rome, Italy: Giuseppe Pugliese, Maria Cristina Ribaudo, Elena Alessi, Martina Vitale, Lorenza Mattia, Chiara Giuliani, Lucilla Bollanti, Francesco G. Conti.
2. Diabetes Unit, Fatebenefratelli San Pietro Hospital, Rome, Italy: Nicolina Di Biase, Filomena La Saracina.
3. Diabetes Unit, Health District, Monterotondo, Rome, Italy: Stefano Balducci, Mario Ranuzzi, Jonida Haxhi, Valeria D’Errico.

***Metabolic Fitness Centers***

1. Department of Human Movement and Sport Sciences, University of Rome ‘Foro Italico’, Rome, Italy: Massimo Sacchetti, Giorgio Orlando.
2. Center for the Study of Metabolism, Rome, Italy: Luca Milo, Roberto Milo.
3. Metabolic Fitness Association, Monterotondo, Rome, Italy: Gianluca Balducci, Enza Spinelli.

**Central laboratory**

Laboratory of Clinical Chemistry, Sant’Andrea University Hospital, Rome, Italy: Patrizia Cardelli, Stefano Cavallo.

**Data Management team**

1. Stefano Balducci, MD, Metabolic Fitness Association O.N.L.U.S., Monterotondo, Rome, Italy; coordinator;
2. Elena Alessi, MD, Diabetes Unit, Sant’Andrea University Hospital, Rome, Italy: responsible for control of data completeness, coherence, and plausibility;
3. Gianluca Balducci, Phisioterapist, Metabolic Fitness Association O.N.L.U.S., Monterotondo, Rome, Italy: responsible for monitoring of adverse events during theoretical and practical counseling sessions;
4. Giorgio Orlando, PhD, Department of Human Movement and Sport Sciences, University of Rome ‘Foro Italico’, Rome, Italy: responsible for estimation of VO_2max_, and analysis of self-reported MS questionnaire;
5. Silvano Zanuso, PhD, Center for Applied Biological & Exercise Sciences, Faculty of Health & Life Sciences, Coventry University, Coventry, UK: responsible for calculation of PA and SED-time from accelerometer readings;
6. Patrizia Cardelli, PhD, Department of Clinical and Molecular Medicine, University of Rome La Sapienza, and Laboratory of Clinical Chemistry, Sant’Andrea University Hospital, Rome, Italy: responsible for biochemical tests;
7. Giuseppe Lucisano, StatD, Center for Outcomes Research and Clinical Epidemiology (CORESEARCH), Pescara, Italy; E-mail: responsible for analysis of well-being/depression and QoL questionnaires.
